# Detection of Genotype 4 Swine Hepatitis E Virus in Systemic Tissues in Cross-Species Infected Rabbits

**DOI:** 10.1371/journal.pone.0171277

**Published:** 2017-01-27

**Authors:** Qiaoxing Wu, Junqing An, Ruiping She, Ruihan Shi, Wenzhuo Hao, MajidHussain Soomro, Xuerui Yuan, Jinling Yang, Jingyuan Wang

**Affiliations:** Laboratory of veterinary pathology and public health, college of veterinary medicine, China Agricultural University, Beijing, China; Centers for Disease Control and Prevention, UNITED STATES

## Abstract

Increasing evidence demonstrates that hepatitis E virus (HEV) can be transmitted across species. According to previous reports, swine HEV has two genotypes, genotype 3 and 4, and both can infect humans by the fecal-oral route. Thus, it is crucial for the control of HEV zoonotic transmission to evaluate the dynamics of viral shedding and distribution in different tissues during cross-species infection by HEV. In this study, rabbits were infected with genotype 4 swine HEV by the intraperitoneal route. The results showed that HEV RNA not only shed in the feces but also in the saliva of some rabbits during infection with swine HEV. Viremia appeared late after infection, and anti-HEV IgG was not obvious until the appearance of high viremia levels. After the rabbits were euthanized, a histopathological examination showed that the livers developed overt hepatitis accompanied by an elevation of alanine aminotransferase (ALT) and aspartate transaminase (AST). Furthermore, HEV RNA was detected in various tissues, especially in the salivary glands and tonsils. Subsequently, negative-stranded HEV RNA was practiced in tissues with positive HEV RNA, which demonstrated that HEV replicated in the tissues. Next, we harvested additional tissues from the liver, salivary gland, tonsil, spleen, thymus gland, lymph node and intestine, which are known as replication sites of swine HEV. Additionally, we also observed the HEV antigen distributed in the organs above through immunohistochemical staining. These results demonstrate that rabbits could be used as an animal model for researching cross-species infection of genotype 4 HEV. It is also noteworthy that HEV can shed in the saliva and presents the risk of droplet transmission. These new data provide valuable information for understanding cross-species infection by HEV.

## Introduction

Hepatitis E (HE) is a fecal-oral transmission disease caused by HEV, which is a non-enveloped, positive-sense, single-stranded RNA virus [1]. Hepatitis E is endemic worldwide and epidemic in developing countries [2, 3]. To date, there are 4 major genotypes of HEV identified in mammals, and avian HEV is associated with substantial liver and spleen disease [3, 4]. Genotypes 1 and 2 are restricted to humans, whereas genotypes 3 and 4 infect humans, pigs and other animal species [2]. The genome of mammalian HEV consists of three open reading frames (ORFs). ORF 1 at the 5’ end encodes non-structural polyproteins. ORF 2 encodes the capsid protein that is the target for vaccine development [2, 5]. ORF 3 encodes a small cytoskeleton-associated phosphoprotein presenting on the suface of virion released from infected cells for viral pathogenesis and release.[3, 6–8].

Animal models have been the main tools for researching HEV due to the lack of an efficient cell culture system [2]. Although cell lines have been developed for culturing some HEV strains [9], practical animal models still play an important role for researching HEV. Pigs, mice and gerbils are good animal models for swine infection [10–12]. In previous studies, animals were infected intravenously with HEV because it was difficult to experimentally reproduce swine HEV infection by the oral route of inoculation in pigs [13, 14]. However, the intravenous route may not be comparable to the natural fecal-oral transmission route because HEV invades the liver by the portal vein, not the hepatic artery, during natural infection. In preliminary studies, rabbits and gerbils have been successfully infected with rabbit HEV and swine HEV, respectively, by intraperitoneal inoculation, which has been regarded as a better route [15–17].

Growing evidence has indicated that hepatitis E is zoonotic. Previous studies showed that HEV can be isolated from rats, boars, rabbits, ferrets, camels and cows [18–24]. Additionally, HEV isolated from pigs and boars can infect humans and result in cross-species infection in zoo-like locations under natural conditions [21, 25, 26]. Birds can be infected with mammalian HEV [26, 27]. Similarly, both swine HEV and rabbit HEV can infect non-human primates experimentally [28, 29]. Recently, it has been demonstrated that infectious HEV can be excreted into milk and infect rhesus macaques, and infectious HEV cannot be inactivated by Pasteurization [24]. HEV-4 had been isolated from the patients with acute hepatitis E and showed high sequence similarity to swine HEV-4 in China[30]. Thus, studying HEV-4 in rabbits is also important because HEV-4 is the predominant genotype in China[30]. In view of public health, it is meaningful to further explore the mechanisms and risks of cross-species infection by HEV.

Therefore, we explored whether rabbits can be infected with genotype 4 swine HEV by the intraperitoneal route and evaluated viral shedding in the feces and saliva. We then detected the levels of antigen, anti-HEV IgG and hepatic enzymes. The rabbits were sacrificed at 28 and 49 days post-inoculation (dpi), and replication sites and the location of the ORF 2 antigen were detected. The goal was to better understand the mechanisms underlying HEV cross-species infection.

## Materials and Methods

### Ethics statement

The animal experiment was approved by the Animal Care and Use Committee of China Agricultural University (CAU) (permit number: 20151110–160). We followed the guidelines of the CAU Animal Care and Use Committee in handling the experimental animals during this study.

### Source and generation of an infectious stock of swine HEV

Strain HB-L3 of swine HEV, belonging to genotype 4 and having 90.9% homology with a Beijing human strain (GenBank No. AJ272108), was isolated and stored by our lab (GenBank No. KJ123761,KX531115) (S1 Table). To generate infectious virus stock, a 10% suspension (w/v in phosphate-buffered saline, PBS) of the stored positive intestinal content was first used to inoculate intraperitoneally two rabbits at 10 mL per day for 7 consecutive days. After 4 weeks post-inoculation (wpi), the fecal samples of both rabbits were positive for HEV RNA. Both rabbits continued feeding for one week and then were euthanized. Samples of livers and intestinal contents were positive for HEV RNA and were stored at -80°C. The viral sequence from the rabbits was homogenous with the stored swine HEV strain. Then, the intestinal contents were prepared in a 10% suspension and used as an infectious viral stock. The titer of the suspension was 6.63×10^7^ genome equivalents per mL.

### Animals

Sixteen 80-day-old female New Zealand white rabbits weighing between 1800 and 2000 g were purchased from the Xing Long Experimental Animal Center, Beijing, China. Before inoculation, the blood and feces of all the rabbits were confirmed to be negative for HEV RNA with reverse transcription-nested PCR (RT-nPCR). The sera were confirmed negative for HEV antigen and HEV antibody using enzyme-linked immunosorbent assay (ELISA).

### Experimental design

Sixteen rabbits were randomly divided into two groups of eight rabbits per group. Each rabbit in the experimental group was inoculated intraperitoneally with 10 mL of viral suspension per day for 7 consecutive days. Each rabbit in the control group was injected with an equal volume of negative fecal suspension. Each rabbit was housed in a separate cage and fed twice a day with access to drinking water. The condition of all the rabbits was monitored everyday, and no rabbit died following viral inoculation. No clinical symptoms were observed in the HEV-infected rabbits. All the rabbits were sacrificed by air-injection at the end of the experiment.

### Sample collection and processing

Throat swab samples and feces were collected and tested for HEV RNA at the indicated times. Blood was collected and tested for HEV antibodies and antigens weekly to avoid animal stress. Half of the rabbits were euthanized at 28 dpi and the other half at 49 dpi. Their blood, bile, hearts, lungs, livers, kidneys, spleens, lymph nodes, thymuses, tonsils, salivary glands, stomachs, small intestines (duodenum, jejunum, ileum), sacculus rotundus (SR), cecum and appendices were collected, stored at -80°C for RT-nPCR and fixed in 4% paraformaldehyde for paraffin sections. The collected serum was tested for alanine aminotransferase (ALT) and aspartate transaminase (AST) levels at the Animal Hospital of China Agriculture University.

### ELISA for HEV antibodies and antigen

All the serum samples were collected and tested for HEV antigen and anti-HEV IgG using an HEV ELISA kit. All assays were performed according to the manufacturer’s instructions (Wantai, Beijing, China)[15, 16].

### RT-nPCR for the detection of HEV RNA and negative-stranded HEV RNA in tissues

Total RNA was extracted from the collected samples using an UltraPure^™^ RNA Kit (CWBIO, Beijing, China); then, the RNA was reverse transcribed using the HiFi script cDNA Synthesize Kit (CWBIO, Beijing, China) according to the manufacturer’s instructions. Nested PCR was carried out to amplify the partial fragments of ORF2 using outer primers P1, P2 and inner primers P3, P4. The PCR parameters were described in Yang’s article [31].

Samples with positive HEV RNA were assayed for negative-sense HEV RNA [17, 28]. The extracted RNA was subjected to cDNA synthesis with the outer primer P1. Parental RNA was degraded using RNaseA, followed by nested PCR with the same PCR parameters. Sterile ddH_2_O was included as the negative control.

### Histopathological examination and immunohistochemical stain for the HEV ORF2 and ORF3 protein

All fixed tissues were processed routinely in paraffin, and 4-μm sections were prepared. The sections were stained with hematoxylin-eosin (H&E) stain for routine histopathological examination. Monoclonal mouse anti-HEV ORF2 and ORF3 antibodies (1:200 dilution; Beijing Protein Innovation, Beijing, China) werethe primary antibody for immunohistochemistry, as described by Mao et al. [16].The samples from the control group were used as the negative control.

## Results

### Dynamic evaluation of HEV RNA shedding in feces and throat swabs

Fecal and throat swab samples from all rabbits in the control group were negative for HEV RNA during the study.

Fecal samples from all rabbits infected with swine HEV were positive for HEV RNA at different time points. At 7 dpi, HEV RNA was first detected in the feces of T4. The feces of all the rabbits were positive for HEV RNA over time. The shedding of HEV RNA was not consecutive in the feces during the early period of infection. Shedding of HEV RNA usually lasts 3~7 days and reappeared after an interval of 7~10 days in the infected rabbits. During the later period of infection, HEV RNA disappeared in the feces of T8 rabbit (Table 1).

**Table 1:** Detection of HEV RNA in the feces and throat of the test group.

Days post-inoculation	No. of rabbits infected by HEV								Total	
	T1	T2	T3	T4	T5	T6	T7	T8	Fecal sample	Throat swab
**1**	-,N	-,N	-,N	-,N	-,N	-,N	-,N	-,N	0/8	0/8
**3**	-,N	-,N	-,N	-,N	-,N	-,N	-,N	-,N	0/8	0/8
**7**	-,N	-,N	-,N	+,N	-,N	-,N	-,N	-,N	1/8	0/8
**10**	-,N	-,N	+,N	-,N	+,N	+,N	-,N	-,N	3/8	0/8
**14**	+,N	-,N	-,N	+,N	-,N	+,N	-,N	+,N	4/8	0/8
**17**	+,N	-,N	-,N	+,N	-,N	-,N	-,N	+,N	3/8	0/8
**21**	-,N	+,N	-,N	+,N	-,N	+,N	+,N	-,N	4/8	0/8
**24**	-,N	+,N	+,N	+,P	-,N	+,N	-,N	-,N	4/8	1/8
**28**	-,N	+,N	+,N	+,P	+,N	-,N	-,N	+,N	5/8	1/8
**31**	NT	NT	NT	NT	+,N	+,N	-,N	-,N	2/4	0/4
**35**	NT	NT	NT	NT	+,N	+,P	-,N	-,N	2/4	1/4
**38**	NT	NT	NT	NT	-,p	+,P	-,N	+,N	2/4	2/4
**42**	NT	NT	NT	NT	-,p	+.N	+,N	-,N	2/4	1/4
**45**	NT	NT	NT	NT	+,P	+.N	+,N	-,N	2/4	1/4
**49**	NT	NT	NT	NT	+,P	+,N	+,N	-,N	3/4	1/4

+/-: positive or negative for HEV RNA in the fecal sample; P/N: positive or negative for HEV RNA by throat swab. The rabbits were inoculated with an HEV suspension from 1 to 7 days. NT: Nos. T1, T2, T3 and T4 were euthanized at 28 dpi.

The throat swab samples from 3 rabbits were positive for HEV RNA during the study. Throat swab samples of T4, T5 and T6 rabbits were positive for HEV RNA at 24 dpi, 35 dpi and 38 dpi, respectively. Shedding of HEV RNA lasted for 3~10 days and disappeared. In the T5 rabbit, throat swab samples were positive for HEV RNA, but the feces were negative for HEV RNA (Table 1).

### Levels of hepatic enzymes after HEV inoculation

**Fig 1:**
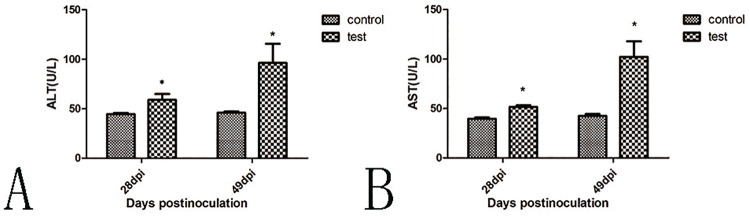
ALT and AST levels at 28 and 49 dpi, respectively. A. The average ALT level was significantly increased in the experimental group compared with the control group (p<0.05) at 28 dpi and 49 dpi. At 49 dpi, the average ALT increased drastically to 96.3 U/L. B. The AST level increased significantly at 28 dpi and 49 dpi (p<0.05). The average AST level reached 102.1 U/L in the experimental group. (*, p<0.05).

At 28 dpi, the ALT and AST levels in the experimental group were significantly higher than in the control group (p<0.05). Dramatic elevation of ALT and AST in the experimental group was observed at 49 dpi. The average ALT and AST reached 96.3 and 102.1 U/L, respectively, in the experimental group (Fig 1). SPSS 20.0 was used for statistical analysis. Paried-sample test was used to calculate the *P* values.

### Detection of HEV antigen and anti-HEV IgG by ELISA

**Fig 2:**
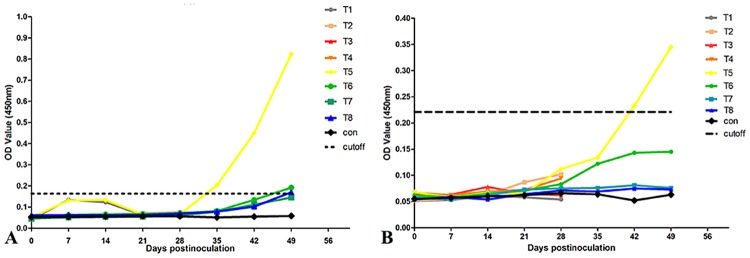
Changes in serum HEV antigen and anti-HEV IgG based on ELISA at various days post-inoculation. A. Serum HEV antigen was first detected in the T5 rabbit at 35 dpi and was elevated until 49 dpi. Serum HEV antigen was also detected in the T6 and T8 rabbits at 49 dpi. B. Anti-HEV IgG appeared in the T5 rabbit at 42 dpi and was elevated until 49 dpi. Other rabbits had no anti-HEV IgG until the end of the experiment. All rabbits in the control group were designated the “control”.

Viremia was first detected at 35 dpi in T5 rabbits and was elevated until 49 dpi. At 49 dpi, viremia was also detected in T6 and T8 rabbits. Anti-HEV IgG was only detected in the T5 rabbit at 42 dpi and remained elevated until 49 dpi (Fig 2). Both HEV antigen and anti-HEV IgG were negative in the control group.

### Distribution of HEV RNA in tissues

All the tissues from rabbits in the control group were negative for HEV RNA throughout the study. At 28 dpi, HEV RNA was detected in various tissues, including liver, bile, salivary gland, spleen, thymus gland, lymph nodes, small intestine, SR, cecum, appendices, and kidney. At 49 dpi, HEV RNA was also detected in the blood, liver, bile, salivary gland, spleen, lymph nodes, small intestine, SR, cecum, appendices, heart, lung, and pancreas (Table 2).

**Table 2:** Distribution of HEV RNA and negative-stranded RNA in various tissues of rabbits infected with swine HEV at the indicated days post-inoculation.

	Positive rate at the indicated days post-inoculation											
	28 dpi						49 dpi					
Tissues	T1	T2	T3	T4	Positive rate	Positive ratio of Negative stranded RNA	T5	T6	T7	T8	Positive rate	Positive ratio of Negative stranded RNA
**Blood**	**-**	**-**	**-**	**-**	**0/4**	**0/4**	**+**	**+**	**-**	**+**	**3/4**	**0/4**
**Bile**	**+**	**+**	**+**	**+**	**4/4**	**0/4**	**+**	**+**	**+**	**+**	**4/4**	**0/4**
**Liver**	**++**	**++**	**++**	**++**	**4/4**	**4/4**	**++**	**++**	**++**	**++**	**4/4**	**4/4**
**Salivary gland**	**-**	**-**	**-**	**++**	**1/4**	**1/4**	**++**	**-**	**-**	**-**	**1/4**	**1/4**
**Tonsil**	**-**	**-**	**-**	**-**	**0/4**	**0/4**	**++**	**-**	**-**	**-**	**1/4**	**1/4**
**spleen**	**-**	**-**	**++**	**-**	**1/4**	**1/4**	**++**	**+**	**-**	**-**	**2/4**	**1/4**
**Thymus gland**	**-**	**-**	**-**	**++**	**1/4**	**1/4**	**-**	**-**	**-**	**-**	**0/4**	**0/4**
**Lymph node**	**-**	**-**	**-**	**+**	**1/4**	**0/4**	**++**	**++**	**-**	**+**	**3/4**	**2/4**
**Stomach**	**-**	**-**	**-**	**-**	**0/4**	**0/4**	**-**	**-**	**-**	**-**	**0/4**	**0/4**
**Small intestine**	**-**	**++**	**++**	**++**	**3/4**	**3/4**	**-**	**+**	**++**	**+**	**3/4**	**1/4**
**SR**	**-**	**++**	**++**	**++**	**3/4**	**3/4**	**-**	**+**	**+**	**++**	**4/4**	**1/4**
**Cecum**	**-**	**++**	**++**	**++**	**3/4**	**3/4**	**++**	**++**	**+**	**+**	**4/4**	**2/4**
**Appendix**	**-**	**++**	**++**	**++**	**3/4**	**3/4**	**+**	**++**	**+**	**+**	**4/4**	**1/4**
**Kidney**	**-**	**-**	**-**	**+**	**1/4**	**0/4**	**-**	**-**	**-**	**-**	**0/4**	**0/4**
**Heart**	**-**	**-**	**-**	**-**	**0/4**	**0/4**	**-**	**-**	**+**	**-**	**2/4**	**0/4**
**Lung**	**-**	**-**	**-**	**-**	**0/4**	**0/4**	**-**	**-**	**+**	**-**	**1/4**	**0/4**
**Pancreas**	**-**	**-**	**-**	**-**	**0/4**	**0/4**	**-**	**-**	**-**	**+**	**1/4**	**0/4**

+: positive for HEV RNA in tissues; -: negative for HEV RNA in tissues; ++: positive for both HEV RNA and negative-stranded RNA.

### Extrahepatic replication of swine HEV in rabbits

The discovery of negative-stranded HEV RNA and ORF 3 antigen in the tissues demonstrates that HEV replicates therein [28,32,33,34]. No negative-stranded HEV RNA was detected in the blood or bile. At 28 dpi, negative-stranded HEV RNA was detected in the liver, salivary gland, spleen, thymus gland, small intestine, SR, cecum and appendices. At 49 dpi, negative-stranded HEV RNA was detected in the liver, salivary gland, tonsil, spleen, lymph nodes, small intestine, SR, cecum and appendices (Table 2). The tissues with negative-stranded HEV RNA were also positive for ORF 3 protein and the distribution of ORF 3 in cells was similar to the ORF 2 (S1 Fig).

### Histopathological examination and immunohistochemistry for HEV antigen in rabbit tissues

**Fig 3:**
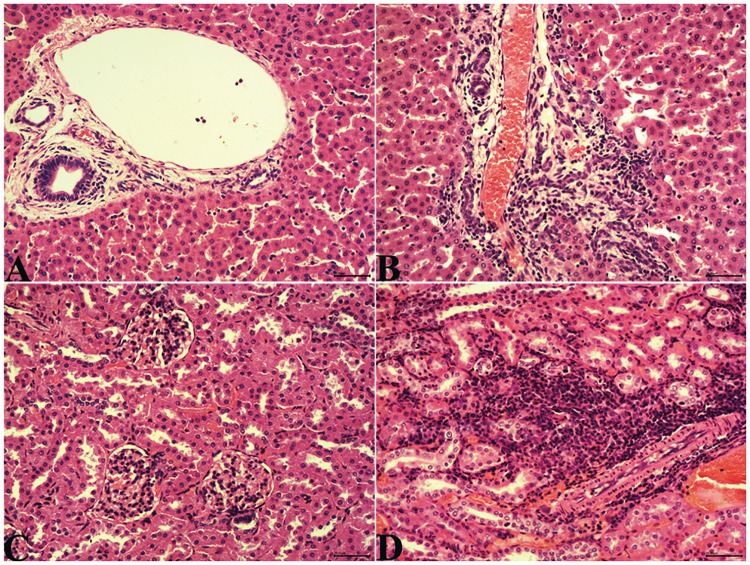
Histopathological analysis of livers and kidneys. A. There were no gross histopathological lesions in the liver sections from the control group. B. A large number of lymphocytes infiltrated the liver, and the bile ducts proliferated in the portal tracts of the liver in the experimental group. C. No gross lesions were observed in the kidney sections from the control group. D. A large number of lymphocytes infiltrated into the mesenchyme of the renal cortex. H&E staining.

Various tissues in the control group revealed no obvious lesions (Fig 3A and 3C). Livers in the experimental group usually revealed multifocal hepatitis characterized by hepatocyte swelling, infiltration of a large number of lymphocytes, and proliferation of the bile ducts in the portal tracts at 28 dpi and 49 dpi (Fig 3B). Additionally, the kidneys also had multifocal inflammation characterized by infiltration of lymphocytes into the mesenchyme of the renal cortex (Fig 3D). Other tissues in the experimental group did not manifest gross histopathological lesions.

**Fig 4:**
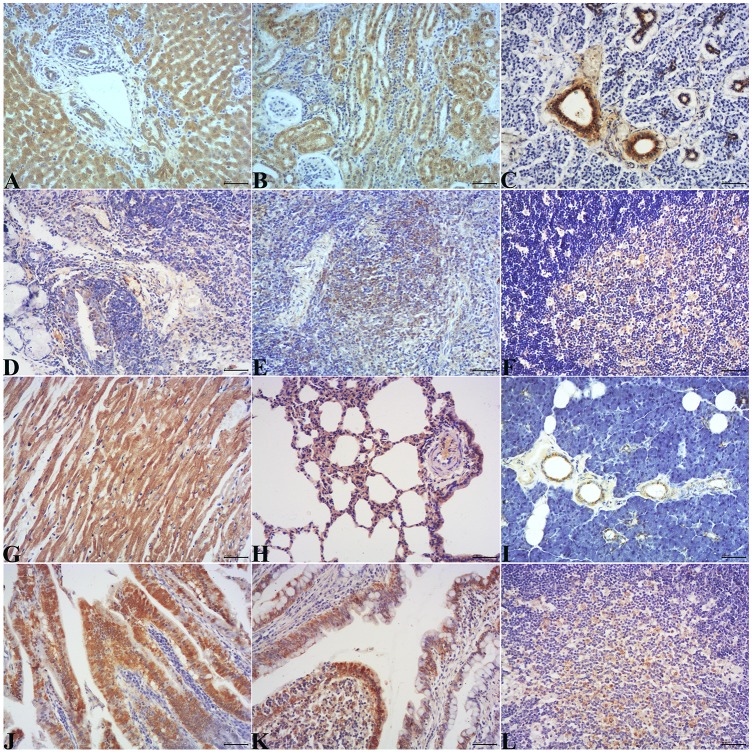
HEV ORF2 antigen localization in various tissues from the experimental group detected using immunohistochemical staining. A. Liver. The positive signal for HEV antigen was detected in hepatocytes and the epithelium of bile ducts. B. Kidney. The positive signal for HEV antigen was detected in the epithelium of the renal tubules. C. Salivary gland. The positive signal for HEV antigen was detected in the duct epithelium of salivary gland. D. Tonsil. The positive signal for HEV antigen was detected in the crypt epithelium and in macrophages in the tonsil. E. Spleen. The positive signal for HEV antigen was detected in macrophages or dendritic cells in the spleen. F. Thymus gland. The positive signal for HEV antigen was detected in thymic epithelial cells in the medulla of the thymus gland. G. Heart. The positive signal for HEV antigen was detected in the myocardium. H. Lung. The positive signal for HEV antigen was detected in the epithelium of the alveoli and bronchioles. I. Pancreas. The positive signal for HEV antigen was detected in the duct epithelium of the pancreas. J. Small intestine. The positive signal for HEV antigen was detected in the mucosal epithelium in the small intestine. K. SR. The positive signal for HEV antigen was detected in the follicle-associated epithelium. L. Appendix. The positive signal for HEV antigen was detected in macrophages or dendritic cells of lymphoid follicles. The positive signal is yellow.

**Fig 5:**
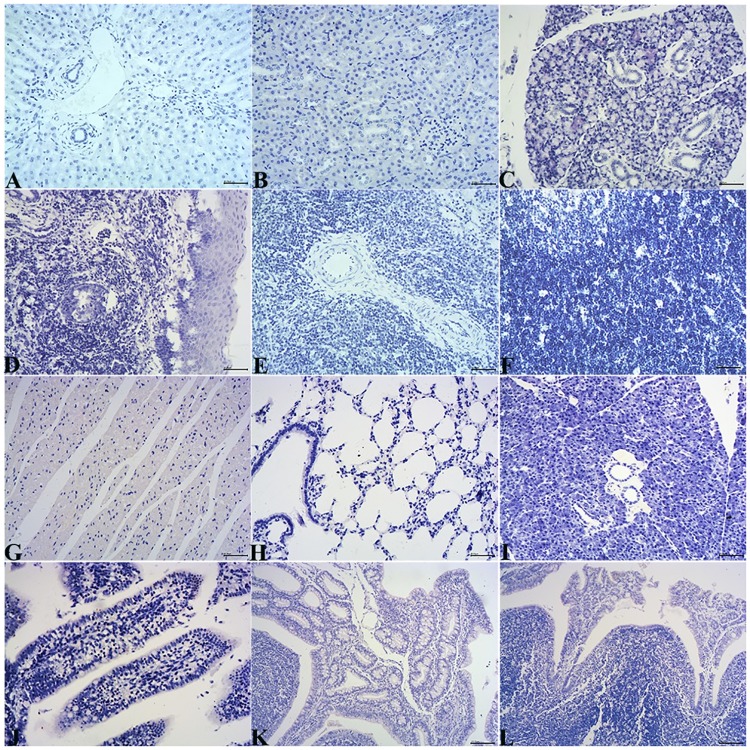
HEV ORF2 antigen localization in various tissues from the control group detected using immunohistochemical staining. No positive signal (yellow) was detected in these tissues of the control group. A-L: liver, kidney, salivary gland, tonsil, spleen, thymus gland, heart, lung, pancreas, small intestine, SR or cecum.

Using immunohistochemical staining, HEV antigen was detected in the liver, salivary gland, tonsil, spleen, thymus gland, lymph nodes, small intestine (duodenum, jejunum and ileum), SR, cecum, appendices, kidney, heart, lung and pancreas. Positive signals were detected in the cytoplasm of hepatocytes and biliary epithelial cells; the duct epithelium of the salivary gland and pancreas; the crypt epithelium and macrophages or dendritic cells of lymphoid follicles in the tonsil, spleen, lymph node, SR, appendix, and intestinal mucosal epithelium; the follicle-associated epithelium of the SR and appendix; the thymic epithelial cells in the medulla, myocardium, the epithelium of the renal tubules and the epithelium of the bronchioles and alveoli (Fig 4). No positive signals were detected in any tissues of the control group (Fig 5).

## Discussion

According to previous reports, rabbits have been infected with swine HEV or genotype 4 HEV and developed hepatitis by intravenous inoculation [5, 32, 33]. However, whether rabbits can be an animal model for researching genotype 4 swine HEV has not been reported. Some studies have shown that intraperitoneal inoculation is a better route to imitate HEV natural infection via the fecal-oral route compared with intravenous inoculation [16]. At the meantime, we also found that the intragastric infection rate of swine HEV-4 in rabbits was obviously lower than intraperitoneal inoculation (unpublished). Additionally, second-passage rabbit HEV challenge with a higher dose of virus results in more severe hepatitis [16, 33]. In our study, we first recovered the swine HEV strain in rabbits and then used the HEV-positive intestinal contents as the inoculum to infect rabbits by continuous intraperitoneal inoculation.

Rabbits infected with swine HEV had obvious HEV shedding in the feces and via throat swabs at different time points. Unlike the duration of fecal viral shedding in pigs and Mongolian gerbils, rabbits infected with swine HEV had intermittent fecal viral shedding in the early stage of infection [12, 15]. However, fecal HEV shedding persisted as time passed. This showed that HEV shedding in the feces may be unstable during the early period of the establishment of HEV infection. In order to better explore HEV shedding during the early period of HEV infection, the interval between sample collections should be shortened. In addition, it is noteworthy that HEV RNA disappeared in the T8 rabbit in the later period of infection, but HEV RNA was detected in the liver, bile and intestine. There are three possible explanations for this. First, the rabbit might enter a latent infection period different from the self-limiting symptoms of HEV infection in humans [34]. The decrease of HEV replication in the intestine may lead to this appearance. Finally, the appearance of intestinal anti-HEV secretory IgA (SIgA) might neutralize HEV to inhibit HEV shedding in the feces during later periods of HEV infection, as seen in other studies [35, 36].

Compared with normal HEV RNA detection in feces, the detection of HEV RNA in throat swab samples is interesting. HEV RNA was once detected in nasal swab materials in pigs experimentally infected with swine HEV, and it was thought that the pig snouts may have been contaminated with feces [12]. Although rabbits are cecotrophs, we do not think the throat swabs were contaminated with feces because not all throat swabs were positive for HEV RNA in rabbits with fecal material positive for HEV. Additionally, the throat swab was positive for HEV RNA in the T5 rabbit, while the fecal samples were negative at 38 dpi and 42 dpi. These results suggest that there was no fecal contamination of the throat swabs. In a previous study, the salivary gland and tonsil were positive for HEV RNA [37]. Therefore, we inferred that the HEV RNA in the throat swab may be from the salivary gland or tonsil. The salivary gland and tonsil were therefore tested for HEV RNA, negative-stranded RNA And ORF3 protein. Negative-stranded HEV RNA is an intermediate product in HEV replication, and the appearance of negative-stranded RNA indicates HEV replication in these tissues [28, 37]. And presence of ORF 3 protein is also indicative of an infectious or replicative viral state[38]. The results showed that HEV RNA existed and replicated in the salivary gland and tonsil in the rabbits with throat swabs positive for HEV, except for the T6 rabbit. Simultaneously, the HEV ORF2 antigen was mainly distributed in the duct epithelium in the salivary gland and the epithelium of the crypts and macrophages in tonsils, as determined by immunohistochemical staining. Considering the structure and function of the salivary gland and tonsil, we hypothesize that HEV can invade and replicate in the salivary gland, and then HEV is shed in the saliva and captured by the tonsil. In view of the fact that HEV in the urine poses a risk of transmission [39], the risk of droplet transmission of HEV should be evaluated further.

Unlike Mongolian gerbils infected with swine HEV [11], rabbits showed a delayed viremia. The viremia usually appeared at 28–42 dpi in pigs with swine HEV and rabbits with genotype 4 HEV [12, 33]. Viremia appeared with the dramatic elevation of ALT and AST levels and resulted in serious lesions in the liver during later periods of HEV infection. The viremia was likely due to the release of HEV in damaged hepatocytes. Additionally, seroconversion was not obvious, except in the T5 rabbit, during this study. Seroconversion appeared at 7 weeks post-inoculation in some pigs infected with swine HEV according to previous reports [11, 12]. It had also been observed that the appearance of seroconversion is later than viremia in other reports [11, 12, 28, 33, 40]. The present study showed that the level of anti-HEV IgG had gradually risen along with the elevation of viremia. Therefore, seroconversion may be dependent on the viral dose of blood in rabbits infected with swine HEV and require a longer period of stimulation.

HEV RNA was detected in various organs, which was consistent with previous reports of animals infected with HEV by experimental or natural infection [10, 16, 22, 28, 33, 41]. However, the existence of HEV RNA did not guarantee viral replication in these organs. Therefore, the detection of HEV negative-stranded RNA and ORF 3 protein was used as evidence of replication [37]. The results showed that the liver, salivary gland, tonsil, spleen, thymus gland, lymph node, small intestine, SR, cecum and appendix were the replication sites of swine HEV in rabbits. These results are similar to research performed in pigs infected with human HEV [37]. The HEV negative-stranded RNA was mainly detected in the liver and intestine and disappeared in the intestine during later periods of infection. This showed that the liver and intestine were the main replication sites, and intestinal replication was temporary during HEV infection. Furthermore, regarding the results of the immunohistochemical staining (ORF2 antigen mainly located in the intestinal epithelium), we considered that HEV is first synthetized in hepatocytes and shed with the secretion of bile, followed by entering and infecting the intestine in experiments using intravenous and intraperitoneal infection. Therefore, this experimental infection was different from natural HEV infection. The replication in the salivary gland was important, owing to HEV shedding with secretion of saliva, as discussion above. Additionally, the spleen, thymus gland and lymph node were also temporary replication sites during HEV infection. The replication of HEV in these tissues needs to be further explored.

In our study, obvious lesions in the liver and kidney were observed, which is consistent with previous studies [39, 41, 42]. Though only one rabbit had HEV RNA in the kidney, HEV ORF2 antigen was detected in the epithelium of the renal tubules without HEV RNA from the experimental group with renal lesions. This showed that the HEV antigen might be taken in by the epithelium of the renal tubules. Whether the kidney injury is related to the immune response to HEV need to be further investigated.

Moreover, HEV ORF2 antigen was mainly distributed in the duct epithelium in the salivary gland, pancreas and mucosal epithelium of the intestine according to immunohistochemical staining. This showed that the epithelium associated with the digestive system might be susceptible to HEV infection. Additionally, HEV ORF2 antigen was observed in macrophages or dendritic cells in the spleen, lymph node, tonsil, gut-associated lymphoid tissue (GALT), SR and appendix [43]. This was related to HEV antigen presentation and the immune response.

## Conclusions

In summary, we demonstrated that rabbits can be infected with genotype 4 swine HEV and develop overt hepatitis. This means that rabbits could be used as a model for infection with genotype 4 swine HEV. Furthermore, detection of HEV shedding in saliva means a risk of droplet transmission, and this may be a new route of HEV transmission which needs further study. These results provide valuable information for the future exploration of the pathogenesis of HEV cross-species infection.

## Supporting Information

S1 Fig HEV ORF3 antigen localization in various tissues from the experimental group detected using immunohistochemical staining. A. Liver. The positive signal for HEV antigen was detected in hepatocytes and the epithelium of bile ducts. B. Salivary gland. The positive signal for HEV antigen was detected in the duct epithelium of salivary gland. C. Tonsil. The positive signal for HEV antigen was detected in the crypt epithelium and in macrophages in the tonsil. D. Spleen. The positive signal for HEV antigen was detected in macrophages or dendritic cells in the spleen. E. Thymus gland. The positive signal for HEV antigen was detected in thymic epithelial cells in the medulla of the thymus gland. G. Small intestine. The positive signal for HEV antigen was detected in the mucosal epithelium in the small intestine. H. SR. The positive signal for HEV antigen was detected in macrophages or dendritic cells of lymphoid follicles. I. Appendix. The positive signal for HEV antigen was detected in the follicle-associated epithelium. The positive signal is yellow.
(DOCX)

S1 Table Homologous analysis of the HB-L3 strain and other genotype 4 HEV strains based on the complete genome. (DOCX)

## References

[1] Reyes GR, Purdy MA, Kim JP, Luk KC, Young LM, Fry KE, et al Isolation of a cDNA from the virus responsible for enterically transmitted non-A, non-B hepatitis. Science. 1990; 4948: 1335–1339. 10.1126/science.2107574 2107574

[2] Meng XJ. Hepatitis E virus: Animal reservoirs and zoonotic risk. Vet Microbiol. 2010; 3–4: 256–265. 10.1016/j.vetmic.2009.03.017 PMC2814965 19361937

[3] Okamoto H. Genetic variability and evolution of hepatitis E virus. Virus Res. 2007; 2: 216–228. 10.1016/j.virusres.2007.02.002 17363102

[4] Payne CJ, Ellis TM, Plant SL, Gregory AR, Wilcox GE. Sequence data suggests big liver and spleen disease virus (BLSV) is genetically related to hepatitis E virus. Vet Microbiol. 1999; 1–2: 119–125. 10.1016/s0378-1135(99)00067-x 10501168

[5] Zhang Y, Zeng H, Liu P, Liu L, Xia J, Wang L, et al Hepatitis E vaccine immunization for rabbits to prevent animal HEV infection and zoonotic transmission. Vaccine. 2015; 38: 4922–4928. 10.1016/j.vaccine.2015.07.040 26212003

[6] Ansari IH, Nanda SK, Durgapal H, Agrawal S, Mohanty SK, Gupta D, et al Cloning, sequencing, and expression of the hepatitis E virus (HEV) nonstructural open reading frame 1 (ORF1) †. J Med Virol. 2000; 3: 275–283. 10630959

[7] Tam AW, Smith MM, Guerra ME, Huang CC, Bradley DW, Fry KE, et al Hepatitis E virus (HEV): Molecular cloning and sequencing of the full-length viral genome. Virology. 1991; 1: 120–131. 10.1016/0042-6822(91)90760-9 PMC7130833 1926770

[8] Huang YW, Opriessnig T, Halbur PG, Meng XJ. Initiation at the third in-frame AUG codon of open reading frame 3 of the hepatitis E virus is essential for viral infectivity in vivo. J Virol. 2007; 6: 3018–3026. 10.1128/JVI.02259-06 PMC1866010 17202216

[9] Jirintai, Mizuo HY, Takagi T, Azuma M, Kusano E, Isoda N, et al Hepatitis E Virus (HEV) strains in serum samples can replicate efficiently in cultured cells despite the coexistence of HEV antibodies: characterization of HEV virions in blood circulation. J Clin Microbiol. 2010; 48: 1112–1125. 10.1128/JCM.02002-09 20107086 PMC2849599

[10] van de Garde MDB, Pas SD, van der Net G, de Man RA, Osterhaus ADME, Haagmans BL, et al Hepatitis E Virus (HEV) Genotype 3 Infection of Human Liver Chimeric Mice as a Model for Chronic HEV Infection. J Virol. 2016; 9: 4394–4401. 10.1128/JVI.00114-16 PMC4836345 26889028

[11] Li W, Sun Q, She R, Wang D, Duan X, Yin J, et al Experimental infection of mongolian gerbils by a genotype 4 strain of swine hepatitis E virus. J Med Virol. 2009; 9: 1591–1596. 10.1002/jmv.21573 19623666

[12] Meng XJ, Halbur PG, Haynes JS, Tsareva TS, Bruna JD, Royer RL, et al Experimental infection of pigs with the newly identified swine hepatitis E virus (swine HEV), but not with human strains of HEV. Arch Virol. 1998; 7: 1405–1415. 10.1007/s007050050384 9722883

[13] Bouwknegt M, Frankena K, Rutjes SA, Wellenberg GJ, Am DRH, Wh VDP, et al Estimation of hepatitis E virus transmission among pigs due to contact-exposure. Vet Res. 2008; 5: 498. 10.1051/vetres:2008017 18367077

[14] Kasorndorkbua C. Pathogenesis and transmission of hepatitis E virus (HEV) in pigs /. Dissertation Abstracts International, Volume: 65–12, Section: B, page: 6232.;Major Professor: Patri. 2004.

[15] Yang Y, Shi R, She R, Soomro MH, Mao J, Fang D, et al Effect of swine hepatitis E virus on the livers of experimentally infected Mongolian gerbils by swine hepatitis E virus. Virus Res. 2015: 171–179. 10.1016/j.virusres.2015.06.007 26093307

[16] Mao J, Zhao Y, She R, Cao B, Xiao P, Wu Q, et al Detection and localization of rabbit hepatitis e virus and antigen in systemic tissues from experimentally intraperitoneally infected rabbits. Plos One. 2014; 3: 1–6. 10.1371/journal.pone.0088607 PMC3942314 24594631

[17] Shi R, Soomro MH, She R, Yang Y, Wang T, Wu Q, et al Evidence of Hepatitis E virus breaking through the blood—brain barrier and replicating in the central nervous system. J Viral Hepatitis. 2016. 10.1111/jvh.12557 27329366

[18] Johne R, Heckel G, Plenge-Bönig A, Kindler E, Maresch C, Reetz J, et al Novel hepatitis E virus genotype in Norway rats, Germany. Emerg Infect Dis. 2011; 10: 1981–1983. 10.3201/eid1609.100444 PMC3294985 20735931

[19] Takahashi M, Nishizawa T, Sato H, Sato Y, Jirintai, Nagashima S, et al Analysis of the full-length genome of a hepatitis E virus isolate obtained from a wild boar in Japan that is classifiable into a novel genotype. J Gen Virol. 2011; 4: 902–908. 10.1099/vir.0.029470-0 21228128

[20] Zhao C, Ma ZT. A novel genotype of hepatitis E virus prevalent among farmed rabbits in China. J Med Virol. 2009; 8: 1371–1379. 10.1002/jmv.21536 19551838

[21] Masuda JI, Yano K, Tamada Y, Takii Y, Ito M, Omagari K, et al Acute hepatitis E of a man who consumed wild boar meat prior to the onset of illness in Nagasaki, Japan. Hepatology Research the Official Journal of the Japan Society of Hepatology. 2005; 3: 178–183. 10.1016/j.hepres.2005.01.008 15792639

[22] Li T, Yang T, Yoshizaki S, Ami Y, Suzaki Y, Ishii K, et al Ferret hepatitis E virus infection induces acute hepatitis and persistent infection in ferrets. Vet Microbiol. 2016: 30–36. 10.1016/j.vetmic.2015.11.014 26790932

[23] Woo PCY, Lau SKP, Teng JLL, Tsang AKL, Joseph M, Wong EYM, et al New Hepatitis E Virus Genotype in Camels, the Middle East. Emerg Infect Dis. 2014; 6: 1044–1048. 10.3201/eid2006.140140 PMC4036782 24856611

[24] Huang F, Li Y, Yu W, Jing S, Wang J, Long F, et al Excretion of infectious hepatitis E virus into milk in cows imposes high risks of zoonosis. Hepatology. 2016. 10.1002/hep.28668 27286751

[25] Christophe Renou J C M B. Possible Zoonotic Transmission of Hepatitis E from Pet Pig to Its Owner. Emerg Infect Dis. 2007; 7: 1094–1096. 10.3201/eid1307.070063 PMC2878240 18214190

[26] ZHANG W, SHEN Q, MOU J, YANG ZB, YUAN CL, CUI L, et al Cross-species infection of hepatitis E virus in a zoo-like location, including birds. Epidemiol Infect. 2008; 08. 10.1017/S095026880700965X PMC2870903 17961279

[27] Li H, Zhu R, She R, Zhang C, Shi R, Wei L, et al Case Report Associated with Aspergillosis and Hepatitis E Virus Coinfection in Himalayan Griffons. Biomed Res Int. 2014; 10: 1–9. 10.1155/2015/287315 PMC4641181 26605326

[28] Liu P, Bu QN, Wang L, Han J, Du RJ, Lei YX, et al Transmission of hepatitis E virus from rabbits to cynomolgus macaques. Emerg Infect Dis. 2013; 4: 559–565. 10.3201/eid1904.120827 PMC3647409 23628346

[29] Meng XJ, Halbur PG, Shapiro MS, Govindarajan S, Bruna JD, Mushahwar IK, et al Genetic and experimental evidence for cross-species infection by swine hepatitis E virus. J Virol. 1998; 12: 9714–9721. 10.1128/jvi.72.12.9714-9721.1998 PMC110481 9811705

[30] Wang L, Liu L, Wei Y, Wang Q, Tian Q, Wang L, et al Clinical and virological profiling of sporadic hepatitis E virus infection in China. J Infection. 2016; 3: 271–279. 10.1016/j.jinf.2016.06.005 27343562

[31] Yang Y, Shi R, She R, Mao J, Zhao Y, Du F, et al Fatal disease associated with Swine Hepatitis E virus and Porcine circovirus 2 co-infection in four weaned pigs in China. Bmc Vet Res. 2015; 1: 1–11. 10.1186/s12917-015-0375-z PMC4379595 25889526

[32] Liu L, Wang L, Xia J, Zhang Y, Zeng H, Liu P, et al Mix-breeding with HEV-infected swine induced inapparent HEV infection in SPF rabbits. J Med Virol. 2016; 4: 681–685. 10.1002/jmv.24374 26334930

[33] Ma H, Zheng L, Liu Y, Zhao C, Harrison TJ, Ma Y, et al Experimental infection of rabbits with rabbit and genotypes 1 and 4 hepatitis E viruses. Plos One. 2010; 2: 1–9. 10.1371/journal.pone.0009160 PMC2820092 20161794

[34] Nanda SK, Ansari IH, Acharya SK, Jameel S, Panda SK. Protracted viremia during acute sporadic hepatitis E virus infection. Gastroenterology. 1995; 1: 225–230. 10.1016/0016-5085(95)90028-4 7806046

[35] Tian DY, Yan C. Significance of serum IgA in patients with acute hepatitis E virus infection. World J Gastroentero. 2006; 24: 3919–3923. 10.3748/wjg.v12.i24.3919 PMC4087946 16804983

[36] M T, S K, H M, K S, K F, K M, et al Simultaneous Detection of Immunoglobulin A (IgA) and IgM Antibodies against Hepatitis E Virus (HEV) Is Highly Specific for Diagnosis of Acute HEV Infection. J Clin Microbiol. 2005; 1: 49–56. 10.1128/JCM.43.1.49-56.2005 PMC540162 15634950

[37] Williams TP, Kasorndorkbua C, Halbur PG, Haqshenas G, Guenette DK, Toth TE, et al Evidence of extrahepatic sites of replication of the hepatitis E virus in a swine model. J Clin Microbiol. 2001; 9: 3040–3046. 10.1128/JCM.39.9.3040-3046.2001 PMC88293 11526125

[38] Bose PD, Das BC, Hazam RK, Kumar A, Medhi S, Kar P. Evidence of extrahepatic replication of hepatitis E virus in human placenta. J Gen Virol. 2014; Pt_6: 1266–1271. 10.1099/vir.0.063602-0 24622580

[39] Geng Y, Zhao C, Huang W, Harrison TJ, Zhang H. Detection and assessment of infectivity of hepatitis E virus in urine. J Hepatol. 2015: 37–43. 10.1016/j.jhep.2015.08.034 26362822

[40] Cossaboom CM, Cordoba L, Sanford BJ, Pineyro P, Kenney SP, Dryman BA, et al Cross-species infection of pigs with a novel rabbit, but not rat, strain of hepatitis E virus isolated in the United States. J Gen Virol. 2012; Pt_8: 1687–1695. 10.1099/vir.0.041509-0 22535776 PMC3541760

[41] Wang L, Xia J, Wang L, Wang Y. Experimental infection of rabbits with genotype 3 hepatitis E virus produced both chronicity and kidney injury. Gut. 2016: 2016–312023. 10.1136/gutjnl-2016-312023 27196597

[42] Soomro MH, Shi R, She R, Yang Y, Hu F, Li H, et al Antigen detection and apoptosis in Mongolian gerbil's kidney experimentally intraperitoneally infected by swine Hepatitis E virus. Virus Res. 2015; 3: 588–593. 10.1016/j.virusres.2015.12.012 26724751

[43] Beyaz F, Ergün E, Bayraktaroğlu AG, Ergün L. The identification of intestinal M cells in the sacculus rotundus and appendix of the Angora rabbit. Vet Res Commun. 2010; 3: 255–265. 10.1007/s11259-010-9349-6 20217227

