# Supplementary material for: Detection of Genotype 4 Swine Hepatitis E Virus in Systemic Tissues in Cross-Species Infected Rabbits
Source: PLoS One. 2017 Jan 27;12(1):e0171277. doi: 10.1371/journal.pone.0171277 (PMC5271373; doi:10.1371/journal.pone.0171277)

**HEV ORF3 antigen localization in various tissues from the experimental group detected using immunohistochemical staining.** A. Liver. The positive signal for HEV antigen was detected in hepatocytes and the epithelium of bile ducts. B. Salivary gland. The positive signal for HEV antigen was detected in the duct epithelium of salivary gland. C. Tonsil. The positive signal for HEV antigen was detected in the crypt epithelium and in macrophages in the tonsil. D. Spleen. The positive signal for HEV antigen was detected in macrophages or dendritic cells in the spleen. E. Thymus gland. The positive signal for HEV antigen was detected in thymic epithelial cells in the medulla of the thymus gland. G. Small intestine. The positive signal for HEV antigen was detected in the mucosal epithelium in the small intestine. H. SR. The positive signal for HEV antigen was detected in macrophages or dendritic cells of lymphoid follicles. I. Appendix. The positive signal for HEV antigen was detected in the follicle-associated epithelium. The positive signal is yellow.


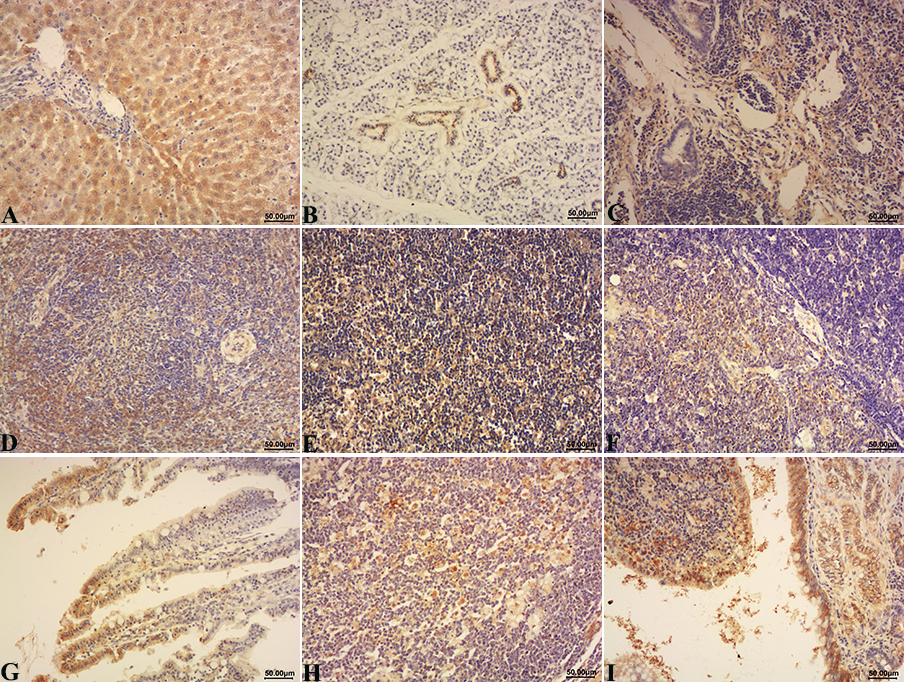

Supplement: S1 Fig — A. Liver. The positive signal for HEV antigen was detected in hepatocytes and the epithelium of bile ducts. B. Salivary gland. The positive signal for HEV antigen was detected in the duct epithelium of salivary gland. C. Tonsil. The positive signal for HEV antigen was detected in the crypt epithelium and in macrophages in the tonsil. D. Spleen. The positive signal for HEV antigen was detected in macrophages or dendritic cells in the spleen. E. Thymus gland. The positive signal for HEV antigen was detected in thymic epithelial cells in the medulla of the thymus gland. G. Small intestine. The positive signal for HEV antigen was detected in the mucosal epithelium in the small intestine. H. SR. The positive signal for HEV antigen was detected in macrophages or dendritic cells of lymphoid follicles. I. Appendix. The positive signal for HEV antigen was detected in the follicle-associated epithelium. The positive signal is yellow. (DOCX) [file pone.0171277.s001.docx]
