# Supplementary material for: Detection of Genotype 4 Swine Hepatitis E Virus in Systemic Tissues in Cross-Species Infected Rabbits
Source: PLoS One. 2017 Jan 27;12(1):e0171277. doi: 10.1371/journal.pone.0171277 (PMC5271373; doi:10.1371/journal.pone.0171277)
Supplement: S1 Table — (DOCX) [file pone.0171277.s002.docx]

S1. Homologous analysis between HB-L3 strain and other genotype 4 HEV strains based on complete genome

| **Strain** |  |  | | **% Identity** | | | | | | | | | | | | | |
| --- | --- | --- | --- | --- | --- | --- | --- | --- | --- | --- | --- | --- | --- | --- | --- | --- | --- |
|  | **1** | **2** | **3** | | **4** | **5** | **6** | **7** | **8** | **9** | **10** | **11** | **12** | **13** | **14** | **15** | **HB-L3** |
| **1** |  | **88.4** | **99.0** | | **84.6** | **84.4** | **84.9** | **83.9** | **84.5** | **84.1** | **84.4** | **86.1** | **86.3** | **84.3** | **84.2** | **84.1** | **84.0** |
| **2** |  |  | **88.5** | | **85.0** | **83.9** | **84.0** | **84.4** | **84.6** | **83.9** | **84.1** | **86.2** | **86.5** | **84.5** | **84.6** | **84.6** | **84.7** |
| **3** |  |  |  | | **84.7** | **84.5** | **84.8** | **84.0** | **84.5** | **84.1** | **84.4** | **86.3** | **86.5** | **84.5** | **84.4** | **84.4** | **84.3** |
| **4** |  |  |  | |  | **84.4** | **84.7** | **84.5** | **92.5** | **84.5** | **84.1** | **84.0** | **84.4** | **84.3** | **839** | **84.4** | **84.4** |
| **5** |  |  |  | |  |  | **85.0** | **84.6** | **84.3** | **84.6** | **89.6** | **83.9** | **84.0** | **89.8** | **89.7** | **89.8** | **89.6** |
| **6** |  |  |  | |  |  |  | **84.3** | **84.6** | **84.3** | **85.0** | **84.3** | **84.4** | **84.4** | **84.9** | **84.9** | **85.2** |
| **7** |  |  |  | |  |  |  |  | **83.3** | **91.7** | **84.4** | **83.4** | **83.7** | **84.4** | **84.7** | **84.7** | **84.7** |
| **8** |  |  |  | |  |  |  |  |  | **83.7** | **83.4** | **83.4** | **83.8** | **83.7** | **83.6** | **83.9** | **83.7** |
| **9** |  |  |  | |  |  |  |  |  |  | **84.1** | **83.6** | **83.8** | **84.4** | **84.5** | **84.5** | **84.8** |
| **10** |  |  |  | |  |  |  |  |  |  |  | **83.8** | **83.9** | **89.3** | **89.0** | **88.9** | **88.6** |
| **11** |  |  |  | |  |  |  |  |  |  |  |  | **95.7** | **83.8** | **83.5** | **83.6** | **83.7** |
| **12** |  |  |  | |  |  |  |  |  |  |  |  |  | **84.2** | **83.7** | **83.9** | **83.9** |
| **13** |  |  |  | |  |  |  |  |  |  |  |  |  |  | **91.3** | **91.3** | **90.9** |
| **14** |  |  |  | |  |  |  |  |  |  |  |  |  |  |  | **96.3** | **95.6** |
| **15** |  |  |  | |  |  |  |  |  |  |  |  |  |  |  |  | **96.6** |
| **HB-L3** |  |  |  | |  |  |  |  |  |  |  |  |  |  |  |  |  |

1 Japan human(AB220978), 2 Japan human (AB080575), 3 Japan human (AB220972), 4 Japan human (AB369690), 5 China Xinjiang swine (AY594199), 6 Indian swine (AY723745), 7 China Heilongjiang Swine(DQ279091), 8 China Shanghai Swine(EF570133), 9 China Guangxi Swine(EU676172), 10 China Gansu Swine(FJ610232), 11 China Xinjiang swine (GU119961), 12 China Hubei Swine(GU188851), 13China Beijing human(AJ272108), 14 China Hubei Swine (GU361892), 15 China Shandong Swine(KF176351). Especially, the homology between HB-L3 strain and Beijing human strain reach 90.9%. The data is from Wei Li’s Master thesis in our lab, 2015.
